# A multidimensional approach to older patients during COVID-19 pandemic: a position paper of the Special Interest Group on Comprehensive Geriatric Assessment of the European Geriatric Medicine Society (EuGMS)

**DOI:** 10.1007/s41999-022-00740-3

**Published:** 2023-01-19

**Authors:** Alberto Pilotto, Carlo Custodero, Katie Palmer, Elisabet Maria Sanchez-Garcia, Eva Topinkova, Maria Cristina Polidori, The Members of the Special Interest Group on Comprehensive Geriatric Assessment of the EuGMS (European Geriatric Medicine Society), Mariana Alves, Mario Barbagallo, Petra Benzinger, Nicolas Berg, Julie Brach, Irwin Cardoso, Maela Caudal, Alberto Cella, Ben Chefi, Annette Ciurea, Ana Maria Cornejo Lingan, Santiago Cotobal Rodeles, Alfonso Cruz-Jentoft, Vito Curiale, Libuse Danielova, Franco Davies, Aafke De Groot, Cathrine De Groot, Jan De Lepeleire, Benjamin De Vries, Anne-Marie Decock, Sanne de Jong, Michael Denkinger, Ayse Dikmeer, Simone Dini, Amaury Durand, Ami Fatin, Marilia Fernandes, Nicola Ferrara, Luigi Ferrucci, Bahaa Francis, Laura Fratiglioni, Ellen Freiberger, Rose Galvin, Blanca Garmendia, Sophie Gillain, Vicky Goodwin, Javier Gomez Pavon, J. A. Goudzwaard, Antonio Greco, Heidi Gruner, Bernd Gunther, Lisa Happe, Vered Hermush, Jan-Kees Huibregtse Bimmel, Ilaria Indiano, Julia Isaak, Javier Jaramillo, Hanna Kerminen, Barbara Kumlehn, Ni Aoife Laocha, Sandra Lau, Lone Lietzen, Isabel Lozano, Ana Teresa Madeira Sarmento, Stefania Maggi, Arduino A. Mangoni, Pedro Marques da Silva, Patricia Mars, Hana Matejovska-Kubesova, Francesco Mattace-Raso, Simone Moeskops, Andrea Molnar, Clarissa Musacchio, Kiruba Nagaratnam, Uomo Nieminen, Margaret O’Connor, Fatma Özge Kayhan Koçak, Marc Paccalin, Anil Palikhe, Tajana Pavic, Raymond Per Nordnes, Izabela Platon, Harmke Polinder, Gabriel Prada, Ragnhild Ragnheim, Lisa Ramsawak, Krzysztof Rewiuk, Carlos Rodrigues, Regina Roller-Wirnsberger, Juhani Rossinen, Giovanni Ruotolo, Georg Ruppe, Dan Ryan, Carlo Sabbà, Elisabet Maria Sanchez-Garcia, Goncalos Sarmento, Sumru Savas, Veronika Schmid, Kaisa Schroderus, Monica Siegrist, Daniel Smedberg, Orla Smit, George Soulis, Maria Tampaki, Natasia Tenkattelaar, Ulrich Thiem, Jorien Tromp, Michiel Van Beek, Lars Van Heijningen, Bob Vandeelen, Heleen Vanderhulst, Nathalie van der Velde, Hana Vankova, Rafaela Verissimo, Nicola Veronese, Filippo Verri, Merel Vonk, Calin Vrabie, Paul Wearing, Michael Weiss, Anna-Karin Welmer, Berenice Werle, Ozlem Ylmaz, Muhammad Shoaib Zaidi, Mihaela Zamfir, Ilo Zanom, Jen Zuidhof

**Affiliations:** 1 Geriatrics Unit, Department of Geriatric Care, Orthogeriatrics and Rehabilitation, Galliera Hospital, Genoa, Italy; 2 Department of Interdisciplinary Medicine, Clinica Medica e Geriatria “Cesare Frugoni”, University of Bari Aldo Moro, P.zza Giulio Cesare, 11, 70124 Bari, Italy; 3 Division of Clinical Geriatrics, Department of Neurobiology, Care Sciences and Society, Karolinska Institutet, Stockholm, Sweden; 4 Servicio de Geriatría, Hospital Universitario Ramón y Cajal, (IRYCIS), Madrid, Spain; 5 Department of Geriatrics, First Faculty of Medicine, Charles University, Prague, Czech Republic; 6 Faculty of Health and Social Sciences, University of South Bohemia, Ceske Budejovice, Czech Republic; 7 Ageing Clinical Research, Department II of Internal Medicine and Center for Molecular Medicine, University of Cologne, Cologne, Germany; 8 Cologne Excellence Cluster on Cellular Stress-Responses in Aging-Associated Diseases (CECAD), University of Cologne, Cologne, Germany

**Keywords:** COVID-19, Comprehensive Geriatric Assessment, Multidimensional frailty, SARS-CoV-2, Prognosis, Decision-making, Intrinsic capacity

## Abstract

**Purpose:**

The COVID-19 pandemic has been a dramatic trigger that has challenged the intrinsic capacity of older adults and of society. Due to the consequences for the older population worldwide, the Special Interest Group on Comprehensive Geriatric Assessment (CGA) of the European Geriatric Medicine Society (EuGMS) took the initiative of collecting evidence on the usefulness of the CGA-based multidimensional approach to older people during the COVID-19 pandemic.

**Methods:**

A narrative review of the most relevant articles published between January 2020 and November 2022 that focused on the multidimensional assessment of older adults during the COVID-19 pandemic.

**Results:**

Current evidence supports the critical role of the multidimensional approach to identify older adults hospitalized with COVID-19 at higher risk of longer hospitalization, functional decline, and short-term mortality. This approach appears to also be pivotal for the adequate stratification and management of the post-COVID condition as well as for the adoption of preventive measures (e.g., vaccinations, healthy lifestyle) among non-infected individuals.

**Conclusion:**

Collecting information on multiple health domains (e.g., functional, cognitive, nutritional, social status, mobility, comorbidities, and polypharmacy) provides a better understanding of the intrinsic capacities and resilience of older adults affected by SARS-CoV-2 infection. The EuGMS SIG on CGA endorses the adoption of the multidimensional approach to guide the clinical management of older adults during the COVID-19 pandemic.

## Key summary points

**Aim:**

To describe the evidence on the usefulness of a Comprehensive Geriatric Assessment (CGA)-based approach during the COVID-19 pandemic.

**Findings:**

The multidimensional, CGA-based approach allows better identification of individual risk profiles and frailty status of infected, recovered but with a post-COVID-19 condition, and non-infected older adults.

**Message:**

Capturing older patients’ needs through CGA may offer the possibility to guide clinical decision and implement personalized medicine.

## Introduction

Compared to previous pandemics in human history (plague, cholera, flu, HIV, SARS, MERS) the SARS-CoV-2 infection tragically affected the older adults population with a mortality rate reaching the share of 75% and over of total deaths [1]. However, aging is characterized by a wide heterogeneity of clinical phenotypes, which reflects different inter-individual intrinsic capacities at the biological level as well as different susceptibility to stressors and injuries. In everyday clinical practice healthcare practitioners face the challenge of managing older patients with similar chronological age and comorbidities but a profoundly different quality of life, everyday functioning and life expectancy. The SARS-CoV-2 infection rapidly revealed this diversity in the older population by highlighting the increasing physiological gap between chronological and biological age [2]. Since early epidemiological findings, it emerged that even though the incidence and mortality rates of SARS-CoV-2 were higher in old compared to young adults [3], chronological age or comorbidity alone did not explain different clinical presentation and course of the disease in the population. Using older chronological age as the only marker of increasing vulnerability not recognizing the wide heterogeneity of older persons may be regarded just as some form of ageism [4]. At the same time, COVID-19 disproportionately affected older and frailer subjects disrupting their homeostatic reserves and leading to poorer, often catastrophic outcomes [4].

Early recognition of symptoms and signs of SARS-CoV-2 infection is crucial not only to contain the spread of contagion but also to increase chances for successful treatments. Thus, several prediction models have been proposed to assist in the prognostication of COVID-19 to support also clinical decision-making [5]. These instruments are mainly based on a few parameters such as age, sex, comorbidities, and COVID-19 disease-specific determinants (e.g., imaging, laboratory, respiratory function markers), and are often characterized by moderate to excellent internal validity. However, these tools subsequently appeared to lack of reproducibility across different cohorts, thus raising concerns about their effective reliability [5].

After almost 3 years of the COVID-19 pandemic, numerous variants of the original virus have already been tracked and new ones are expected to occur [6]. However the widespread vaccination programs and the introduction of antivirals and monoclonal antibodies therapies have changed the shape of pandemic, drastically reducing the fatality, the hospitalization and intensive care unit (ICU) admission rate and also the cases incidence [6, 7]. In this transition toward an endemic phase of COVID-19 greater efforts should be reserved for the long-term consequences of the infection and indirect effects of the pandemic among older adults.

From a geriatric, more comprehensive, perspective, the COVID-19 crisis clearly unmasked the fundamental role of frailty, as surrogate indicator of biological age, versus that of chronological age, to highlight the risk for poor disease trajectories in older adults [2, 8]. Due the main and unique characteristics of this infection in older adults such as: (1) the atypical manifestations, (2) the systemic involvement, (3) the frequent long-term course of the disease, (4) the indirect effects on psycho-physical health, the COVID-19 disease is often referred as a kind of geriatric syndrome that, as the other ones, potentially might benefit from a multidimensional approach. The Special Interest Group on Comprehensive Geriatric Assessment (CGA) of the European Geriatric Medicine Society (EuGMS) took the initiative of collecting evidence on the benefits of the CGA-based multidimensional approach to older people during the COVID-19 pandemic.

## A multidimensional approach to the frail older adults affected by COVID-19

Frailty is characterized by decreased functional reserve and reduced capacity to cope with stressors, and predisposes individuals to a higher risk of mortality, hospitalization, falls, and institutionalization, among others [9]. In line with the pathophysiology of frailty and its typical features in older patients with other age-related diseases, frail older adults affected by COVID-19 commonly experience atypical presentation of the disease [10]. Typical manifestations of a respiratory infection such as fever, cough, and dyspnea, in COVID-19 disease are often paralleled by systemic involvement with gastrointestinal, neurological, cardiovascular, and dermatological manifestations [11]. Moreover, there are signs and symptoms which occur more frequently depending on the COVID-19 variant [11]. In older adults, COVID-19 often behaves differently with the commonly recognized manifestations that are further blunted and hidden by non-specific symptoms including hypotension, sudden functional decline, falls, and delirium [10, 12–14]. Accordingly, the prevalence of typical COVID-19 clinical features has been shown to be low in frail participants [10]. For example, as for other infectious diseases occurring in older adults, fever was significantly less reported in frail COVID-19 patients independent of chronological age and its presence was associated with better chances of survival [10]. On the other hand, it has been shown that frailty is associated with greater COVID-19 severity and poorer prognosis, including longer length of hospital stay, higher incidence of admission to ICU, need for invasive mechanical ventilation, delirium, and mortality [15, 16]. Therefore, it quickly became clear that prompt identification of frailty is crucial to guide clinical decision-making for older patients with COVID-19.

Despite this evidence, profound discrepancies emerged among the clinical values of the main constructs of frailty—i.e., functional, biological (phenotypic) or deficit accumulation models [17]. Previously, the agreement between different theoretical models of frailty had proven extremely limited (roughly 3%), due to the fact that different frailty tools obviously capture different groups of frail persons [17]. Consistently, data from the UK Biobank covering the records of 383,845 older adults showed that frailty, both assessed by means of the deficit accumulation (Frailty Index) [18] and of the biological model (Fried criteria) [19], was associated with two times higher risk of severe COVID-19 disease, but the proportion of frail patients according to each of the two definitions was remarkably different [20]. Indeed, an agreement on the presence of frailty was reached only in 1% of participants [20].

To complicate this already convolute picture, rapid screening tools for frailty have been adopted such as the Clinical Frailty Scale (CFS), a 9-point scale in which a score from 1 “very fit” to 9 “terminally ill” is based on clinical judgement of performance status [21]. The CFS has been endorsed by the National Institute for Health & Care Excellence (NICE) guidelines as a practical tool useful to differentiate COVID-19 patients who might not benefit from admission to ICU (those with CFS ≥ 5) [22]. In a meta-analysis including 3817 patients from seven studies, it has been demonstrated that the increase of CFS is linearly correlated with higher mortality risk from SARS-CoV-2 infection, with a 12% higher risk for each one-unit increase of the score [23]. However, several authors questioned the role of simple tools as they do not adequately characterize frailty status and, therefore, cannot significantly assist in clinical management [8, 24, 25]. Aspects of health like comorbidities, polypharmacy, nutrition, cognition, mobility, independence in activities of daily living, and social conditions are not captured by “simple” tools although they are valid, established, unavoidable factors needed for the identification process of the individual’s risk profile [26]. In other words, “simple” tools are unable to grasp “complexity” which is typical of the ageing process itself and its transition into frailty. The latter can be adequately diagnosed by accurate evaluation of physical, functional, nutritional, and psycho-social domains known to influence individual prognosis. The gold standard in the clinical evaluation of health status of older adults is the Comprehensive Geriatric Assessment (CGA) [27, 28]. A standardized CGA could offer a real picture of biological ageing [29]. Mounting evidence supports the usefulness of CGA-based interventions in different settings for reducing multiple negative outcomes including the risk of institutionalization at discharge in hospitalized older adults [30].

## The multidimensional approach, prognosis, and clinical decision making

The experience of the COVID-19 pandemic strengthened the relevance of the multidimensional approach. In a large observational study, Lee et al. explored predictors of 30-day mortality among 64,733 long-term care residents during the first COVID-19 pandemic wave in Canada [31]. The best predictors of mortality in older adults who tested positive for SARS-CoV-2 infection were activities of daily living (ADLs), cognitive performance, levels of social engagement, and presence of/or risk of developing pressure sores [31], all components traditionally evaluated by a standard CGA. Consistently, in a United States cohort of 5256 nursing home residents with SARS-CoV-2 infection, male sex, age older than 80 years, comorbidities (i.e., diabetes and chronic kidney disease), cognitive and functional impairment were associated with a higher risk of 30-day mortality [32]. Moreover, in a large Korean population-based study, it has been shown that people aged 60 and older who engaged in aerobic and muscle-strengthening activities before the pandemic, had a significantly lower risk of experiencing severe COVID-19 disease, as defined by the need for supplemental oxygen or invasive ventilation, ICU admission, or death [33]. As these data support the concept that multidomain information might offer a better picture of frailty status than mono- or oligo-dimensional models, recently the “multidimensional model of frailty” was proposed to highlight the pivotal role of the CGA for the accurate identification of frailty [26].

Currently, few frailty instruments are specifically built on information derived from a standard CGA. Among these we can consider the following as multidimensional-based: the Geriatric 8 frailty questionnaire (G8) [34], the Edmonton Frailty Scale (EFS) [35], and the Multidimensional Prognostic Index (MPI) [36]. The MPI was the only CGA-based tool (exploring functional, cognitive, nutritional status, mobility, multi-morbidity, number of drugs, and co-habitation status) extensively validated and implemented in older adults with different clinical conditions and in different settings (e.g., hospital, nursing home, community, and general practice) [26].

Among the older population, the prevalence of frailty based upon the calculation of the MPI (high-risk category: MPI-3) has been estimated as 26.8%, varying widely according to the clinical setting (i.e., 13.3% in the community, 29.8% in hospital, and 51.5% in nursing homes) [37]. Evidence preceding the COVID-19 pandemic showed that the MPI was able to predict short-term mortality among older inpatients with community-acquired pneumonia [38]. Also, the MPI might guide clinical decisions in older adults with acute respiratory failure; indeed higher values (above 0.83, range from 0 = no risk to 1 = higher risk of negative outcomes) were associated with a higher risk of non-invasive ventilation failure and, therefore, should discourage unnecessary delay in intubation procedures [39].

Recent findings confirmed that MPI is a reliable prognostic indicator also in older adults with COVID-19 disease [40–44]. Frailer subjects hospitalized for COVID-19 disease reported more atypical presentation (e.g., delirium), and had longer hospitalization, greater functional decline, and a six-time higher risk of in-hospital mortality [40, 42]. A prospective, multicenter study of the SIG-on-CGA of the EuGMS among 502 older people hospitalized for COVID-19 in 10 European hospitals reported that mechanical ventilation was associated with a higher risk of rehospitalization and/or mortality during the 90-day follow-up period (hazard ratio 1.56, 95% CI 1.09–2.23) in frailer participants (MPI values > 0.50), but not in not-frail participants [43]. The accuracy of the model including age, sex, respiratory parameters, and MPI was good (AUC = 0.783) with a highly significant Net Reclassification Index (0.2756, *p* < 0.001) and Improvement Discrimination Index (0.1858, *p* < 0.001), indicating good discrimination of the model in predicting outcomes [43]. Thus among hospitalized older adults we surely have to consider clinical criteria for mechanical ventilation (e.g. SpO2, P/F, respiratory rate), as for other potential treatments, together with the individual patient prognosis based on CGA instruments, but not taking in account age as a mere disqualification criterion.

Also among 3946 older adults residents in long-term facilities incidence rate of death in people with positive COVID-19 swab was more than doubled than in those negative and was progressively higher by MPI category (from 2.57 per 1000 persons-days in the MPI-1 class to 3.11 in the MPI-2 class and 3.33 in the MPI-3 class) [41]. Finally, data from community-dwelling older population showed that subjects with multidimensional prefrailty/frailty (MPI-2 and 3 classes) before the COVID-19 pandemic, had higher rates of hospitalization and SARS-CoV-2 infection compared to robust ones (MPI-1 class) [44]. Frailer subjects affected by COVID-19, during 12 months of follow-up, had more than the four-time higher risk of experiencing further worsening of frailty condition compared to robust and non-infected subjects [44].

Seminal experiences on the application of a CGA-based approach, in the management of acute disease (including SARS-CoV-2 infection) or exacerbation of a chronic condition in multimorbid older adults in a home care setting (hospital-at-home model) during the COVID-19 pandemic, showed this model was feasible and acceptable and has a high potential for further development both for the step-up (community-dwelling patients avoiding hospitalization) and the step-down (patients early discharged from the hospital) [45].

A CGA-based approach could improve the management of older adults with COVID-19 first of all capturing and monitoring the domains with greater impairment, but also allowing to set personalized care plans in different settings (e.g., home care support, need for hospitalization, invasive or non-invasive ventilation) taking into account also the prognosis and the needs of the patients and their caregivers.

## The multidimensional approach to older patients affected by the post-COVID-19 condition

Another emerging issue is the long-lasting sequelae of COVID-19 disease also after virological clearance of SARS-CoV-2, the so-called “post-COVID-19” or “long-COVID-19” condition [46, 47]. The WHO has defined this new nosological entity as a condition characterized by the persistence of symptoms (e.g. chest pain, dyspnea) or new ones (e.g. fatigue, cognitive disorders), not explained by alternative diagnosis, occurring after 3 months from the onset of COVID-19 and lasting at least 2 months [48]. It is already well-known that the effects of cytokines storm, fueling inflammaging process, and prolonged hospitalization and ICU stay may lead to post-intensive care syndrome (PICS) appearing as a poorly reversible cognitive, psychological, and physical disability for 6–12 months after hospital discharge [49]. Indeed having had COVID-19 may generate, for example, a state of poorly reversible psychological distress in particular among older women [50]. However, for COVID-19 also persons not hospitalized or pauci-symptomatic during the infectious phase can experience long-term consequences [51]. Epidemiologic findings have shown that older adults are more prone to develop this condition [52], with an estimated prevalence of about 10% among older adults hospitalized with acute COVID-19 disease [53]. Post-COVID-19 condition is frequently characterized by multiorgan involvement that may require a multidisciplinary team (including internist, pneumologist, infectivologist, radiologist, cardiologist, gastroenterologist, neurologist, ophthalmologist, physiatrist) needing the coordination by a geriatrician [54]. As shown in a cohort of COVID-19 survivors followed 90 days after ICU discharge, new onset frailty or its further progression toward disability might be potential common manifestations of post-COVID-19 condition among older adults [55]. Evidence of a multidimensional involvement in post-COVID-19 condition with cognitive, functional, psychological impairment [52, 53, 56] further supports the potential of a CGA-based approach in the management of these patients. For post-COVID-19 patients CGA may allow an holistic approach useful to set early and tailored plans for effective rehabilitation and follow-up [57].

## The multidimensional approach to older individuals during a pandemic

The consequences of the COVID-19 pandemic reach far beyond the direct risks related to SARS-CoV-2 infection. Excess mortality during the first and second pandemic waves was estimated around 15% but was not fully attributable to SARS-CoV-2 infection [58]. Also infection-control measures might have undermine older individuals’ health in different ways [59], leading to an excess of mortality indirectly related to COVID-19, for example by affecting: (i) the management of patients with already existing noncommunicable diseases (NCDs) such as controlling symptoms and disease progression; (ii) NCD risk factors (lifestyle factors, blood pressure, physical exercise, and weight, etc.) and consequences on future NCD development; (iii) other independent predictors of morbidity and mortality such as functioning, cognitive performance, vitamin D levels, loneliness, wellbeing, psychiatric disorders and symptoms.

In particular, it seems that the excess of deaths not directly related to SARS-CoV-2 infection was significantly higher among persons older than 80 years, accounting for 12–17% of the total excess of mortality [60]. For example, among nursing home residents, during a median follow-up of 275 days, multidimensional frailty (MPI-3) and prefrailty (MPI-2) were associated with a 54% and 89% increase in mortality risk compared to the MPI-1 class, independently of SARS-CoV-2 infection [41]. First of all, restriction measures during total lockdown and fear of contagion led to a consistent loss of healthcare assistance with reduced follow-ups for NCDs, poorer compliance to therapies, and higher risk of sudden death [61–63], particularly among more vulnerable older adults. Secondarily, the parallel spread of poor lifestyle habits (e.g., inadequate nutritional intake, binge eating, alcoholism, smoke, and physical inactivity) and the increase of other risk factors such as loneliness, poor functioning, subjective cognitive complains led to a significantly higher risk of depressive and cognitive disorders, obesity, sarcopenia, falls and an expected further rise in the near future of NCDs [64]. A Finnish study showed that older persons with increasing age and those living alone appeared to be more susceptible to negative lifestyle changes during the pandemic [65] and a Spanish study demonstrated that those who undertook regular, moderate-vigorous exercise during quarantine reported higher scores in resilience and lower depressive symptoms [66]. Being female, living alone, and having four or more NCDs were independently associated with increased loneliness in a study on older adults with multimorbidity [67] and lacking an open space at home was associated with more negative feelings in older persons in Spain [68]. Personality traits (greater intellect, emotional stability, and extraversion) and having a higher general cognitive ability were found to be associated with more positive changes in psychosocial and behavioral factors during the lockdown in a Scottish cohort whereas individuals with a history of cardiovascular disease or who lived alone were more likely to experience negative changes [69]. Greater levels of multidimensional frailty have been correlated to a higher risk of psychiatric disorders and falls in community-dwelling-older adults during the COVID-19 pandemic [70] and further progression of frailty condition at 1-year follow-up also independently by COVID-19 positivity [44].

Although these are just a few examples, they emphasize the need to ensure a comprehensive evaluation of multiple factors to stratify people at higher risk of negative outcomes and plan interventions to counteract the negative consequences of the pandemic. For example, CGA-based telemedicine [71], self-management programs for chronic diseases [72], physical activity programs (including both aerobic and muscle strengthening exercise) [33], hospital-at-home interventions [45] may represent useful strategies for better management of frail older patients avoiding unnecessary hospitalizations.

Moreover, in terms of preventive measures, vaccination coverage drastically reduced COVID-19 incidence, emergency department visits, and hospitalization rate among older adults [73, 74], but it is only one piece of the puzzle for the “exit strategy” from the COVID-19 pandemic. Even in this context, the multidimensional approach could be helpful to explore more compromised domains of health status and design ad hoc interventions [72, 75]. Reinforcement and promotion of healthy lifestyles (e.g. physical activity programs, balanced diet, cognitive training, socialization activities) not only could slow down the aging process and improve quality of life [76, 77], but could also promote better response to vaccines [78].

## EuGMS SIG on CGA statements

There is still a paucity of well-designed randomized controlled studies testing the effect of CGA-based interventions to face the direct and indirect effects of COVID-19. In the view of adopting a multidimensional approach for the management of older patients during the COVID-19 pandemic, further evidence should be gathered in the research field and the following milestones of the geriatric evaluation should be incorporated into clinical practice:

**Table:** 

EuGMS SIG on CGA statements for CGA-based approach during COVID-19 pandemic
Use of CGA should be promoted for routine assessment of biological age and frailty
CGA may help in the identification of atypical presentation, and signs and symptoms of acute and long-term consequences of SARS-CoV-2 infection
CGA may unmask risk factors for COVID-19 disease soliciting adoption of SARS-CoV-2 preventive measures (e.g., vaccinations, healthy lifestyle)
CGA-based assessment of multidimensional frailty should guide clinical decision-making in older adults with COVID-19 (e.g., home care support, need for hospitalization, need for invasive or non-invasive ventilation)
CGA approach is useful to set early and tailored plans for effective rehabilitation and follow-up of older subjects with the post-COVID-19 condition
CGA may support tailored clinical care management programs in community-dwelling older adults during the COVID-19 pandemic (e.g. telemedicine, self-management programs for chronic diseases, hospital-at-home)
SIG on CGA of EuGMS calls for a multidimensional approach to older people in both geriatric and non-geriatric settings

## Data Availability

This article is not based on analysis of specific data.
